# Programmed Cell Death-Ligand 1 Expression Status in Urothelial Carcinoma According to Clinical and Pathological Factors: A Multi-Institutional Retrospective Study

**DOI:** 10.3389/fonc.2020.568809

**Published:** 2020-10-02

**Authors:** Hyung Suk Kim, Won Sik Jang, Won Sik Ham, Seung Il Jung, Dong Hyun Lee, Ja Hyeon Ku, Hong Koo Ha, Ja Yoon Ku, Se Young Choi, In Ho Chang, Taesoo Choi, Wan Song, Seong Soo Jeon, Byong Chang Jeong, Sung Han Kim, Ho Kyung Seo

**Affiliations:** ^1^Department of Urology, Dongguk University Ilsan Medical Center, Dongguk University College of Medicine, Goyang, South Korea; ^2^Department of Urology, Urological Science Institute, Yonsei University College of Medicine, Seoul, South Korea; ^3^Department of Urology, Chonnam National University Hwasun Hospital, Chonnam National University Medical School, Hwasum, South Korea; ^4^Department of Urology, Ewha Womans University Mokdong Hospital, Ewha Womans University School of Medicine, Seoul, South Korea; ^5^Department of Urology, Seoul National University Hospital, Seoul National University College of Medicine, Seoul, South Korea; ^6^Department of Urology, Pusan National University Hospital, Pusan National University School of Medicine, Busan, South Korea; ^7^Department of Urology, Chung-Ang University Hospital, Chung-Ang University College of Medicine, Seoul, South Korea; ^8^Department of Urology, Kyung Hee University Hospital at Gangdong, Kyung Hee University School of Medicine, Seoul, Korea; ^9^Department of Urology, Samsung Medical Center, Sungkyunkwan University School of Medicine, Seoul, South Korea; ^10^Department of Urology, Center for Urologic Cancer, Hospital and Division of Tumor Immunology, Research Institute, National Cancer Center, Goyang, South Korea

**Keywords:** urothelial carcinoma, immune cell, programmed cell death-ligand 1, immunohistochemistry, immune checkpoint

## Abstract

**Purpose:** To investigate programmed cell death-ligand 1 (PD-L1) expression status and the clinical and pathological factors related to its expression in urothelial carcinoma (UC) patients.

**Materials and Methods:** Data from 761 UC patients who underwent testing for PD-L1 expression using the VENTANA (SP-142 immunohistochemistry assay) for measuring PD-L1 expression according to the manufacturer's protocol between February 2016 and July 2019 were retrospectively reviewed. Patients were categorized into three groups based on the percentage of tumor area covered by PD-L1-expressing tumor-infiltrating immune cells (ICs) as follows: IC0 (<1%), IC1 (≥1% and <5%), and IC2/3 (≥5%). Positive PD-L1 expression was defined as IC2/3 (≥5%). The factors related to positive PD-L1 expression were assessed by using unadjusted and adjusted logistic regression analyses.

**Results:** In the entire cohort, 213 (28%) patients showed positive PD-L1 expression. Final adjusted regression analyses for positive PD-L1 expression revealed that several factors, including intravesical BCG prior to PD-L1 testing (odds ratio [OR] 0.57, 95% confidence interval [CI] 0.37–0.96), advanced tumor stage (stage III/IV) (OR 2.04, 95% CI 1.41–2.93), and high tumor grade (OR 5.31, 95% CI 2.38–11.83) were significantly associated with positive PD-L1 expression.

**Conclusions:** This study showed that the PD-L1 expression is associated with several clinical and pathological factors for the first time in a real-world setting. Further follow-up clinical trials should consider adjusting these factors, including intravesical BCG treatment, tumor stage and grade to clarify the utility of PD-L1 as a biomarker.

## Introduction

Immune checkpoints, such as programmed cell death-1 (PD-1) and its associated ligand (PD-L1), have attracted significant attention as major protein targets for systemic immunotherapy of a number of solid tumors, including urothelial carcinoma (UC) (1). In the tumor microenvironment, tumor cells use these immune checkpoints to evade immune system attack by blocking T-cell function. Therefore, anti-tumor immunity can be achieved by restoring T-cell function using antibodies that inhibit the receptor-ligand interaction and block the immune checkpoints (1). Considering the immunogenic features of UC, there have been a number of clinical trials of immune checkpoint inhibitors (ICI) for UC in recent years (2–9). On the basis of the efficacy and safety identified in these clinical trials, five PD-1(pembrolizumab, nivolumab)/PD-L1(atezolizumab, durvalumab, and avelumab) inhibitors have been recommended for first or second-line use in the treatment of locally advanced or metastatic UC by international guidelines (10).

However, approximately 70–80% of patients are unresponsive to ICI (1). Therefore, biomarkers for patient response would be useful for identifying the optimal patient population which is most likely to benefit from treatment with ICI. Several biomarker candidates have been explored in relation to ICI therapy in UC (11–14). Among these, PD-L1 expression has been suggested as a potential biomarker for predicting the response to and prognosis following ICI therapy in UC patients (2–9, 15–23). However, previous studies have reported contradictory results, even with the same drug and methodology. For example, the KEYNOTE-045 study of pembrolizumab found no association between objective response rate and combined positive score (CPS) for PD-L1 expression, but the KEYNOTE-052 study did in first line (2, 8).

To date, four distinct immunohistochemistry (IHC) assays are available for measuring PD-L1 expression in UC (24–26). There are disparities among the IHC assays for PD-L1 expression in terms of the cell populations assayed (tumor cells [TCs] or tumor-infiltrating immune cells [ICs]), the type of detection antibodies used, and the cutoff values for scoring, making it difficult to generalize their results (24–27). In addition, PD-L1 expression test results can be affected by multiple factors, including specimen size, biopsy location, timing of tissue collection, and treatment modality (i.e., radiation, chemotherapy, and intravesical instillation of bacillus Calmette–Guerin [BCG]) (11, 24–28).

In the present study, we aimed to identify the PD-L1 expression status using the VENTANA (SP142) test which was used as a companion diagnostic test for atezolizumab in Korea and the factors significantly associated with positive PD-L1 expression.

## Materials and Methods

### Study Population

This retrospective multicenter study's protocol was approved by the Institutional Review Board of each participating institution (approval no. of corresponding author NCC-2019-0080). The requirement for written informed consent was waived because of the retrospective design. All patient data and records were anonymized before the analysis. All study protocols were complied with the principles of the Declaration of Helsinki. We initially reviewed the medical records of patients with UC histologically confirmed through a variety of surgical procedures, such as transurethral resection (TUR), cystectomy, nephroureterectomy or ureterectomy, and biopsy, between February 2016 and July 2019 at nine institutions. Among these, we included UC patients who received an IHC diagnostic assay for PD-L1 expression and excluded those with pure adenocarcinoma, pure squamous cell carcinoma and small cell carcinoma.

### IHC Assay for PD-L1 Expression

To avoid heterogeneities due to differences between IHC diagnostic assays for PD-L1 expression (24–26), we only included patients who received VENTANA testing, which uses SP142 as a detection antibody for measuring PD-L1 expression in tumor-infiltrating ICs according to the manufacturer's protocol. It was originally designed to identify patients who are likely to respond to atezolizumab treatment which, in the past, was only reimbursed by the government for second line therapy in metastatic UC in Korea based on the results of the VENTANA test. In reference to previous reports on atezolizumab (3, 4, 9), PD-L1 expression level was initially classified into the following three groups based on the percentage of the tumor area covered by PD-L1 expressing ICs as measured by the VENTANA SP142 IHC assay: IC0 (<1%), IC1 (≥1 and <5%), and IC2/3 (≥5%). For subsequent analyses, PD-L1 expression was dichotomized as positive (≥5%) or negative (<5%) using a 5% cutoff value. The results were interpreted by the urologic pathologists belonging to each institution after completion of special training proposed by Roche.

### Acquisition and Definition of Variables

A number of variables evaluated at the time of tissue biopsy were examined to identify any relationships with positive PD-L1 expression. The clinical variables included in the analysis were age (<70 vs. ≥70 years), sex, smoking status, the presence or absence of intravesical treatments (mitomycin-C, BCG) and systemic chemotherapy prior to PD-L1 testing, and the presence or absence of metastasis at the diagnosis of UC. PD-L1 assay-related variables included organ type (lower tract, bladder or urethra/upper tract, renal pelvis or ureter/metastatic site), operation type (TUR/cystectomy, radical or partial/nephroureterectomy, or ureterectomy/biopsy for a primary or metastatic lesion), pathologic tumor stage (stage I/II/III/IV), pathologic tumor grade (low vs. high grade), and time between tissue acquisition and PD-L1 testing (<1 vs. ≥1 month). Tumor stage and grade were assigned according to the 2010 American Joint Committee on Cancer staging system and the 2004 World Health organization system, respectively. For efficient analysis, tumor stage was categorized into two groups: organ confined (stage I/II) and non-organ confined (stage III/IV) disease.

### Statistical Analysis

Continuous variables are expressed as the median and interquartile range (IQR) and categorical variables are expressed as absolute numbers and relative percentages. The relationships between the included variables and PD-L1 expression were assessed using chi-squared test in the case of categorical variables. The significant factors associated with positive PD-L1 expression were evaluated using unadjusted and adjusted logistic regression analyses. All analyses were conducted with SPSS version 21.0 (SPSS Inc., Chicago, IL, USA) and two-sided *P*-values of < 0.05 were considered to be statistically significant.

## Results

### Baseline Characteristics of the Study Population

A total of 761 UC patients consisting of between a minimum of 8 and a maximum of 297 subjects per institution were eligible for analysis. Table 1 summarizes the distribution of the parameters in the entire study cohort (*n* = 761) and each subgroup (negative and positive PD-L1) divided based on the 5% cutoff value. The study cohort had a median age of 70 (IQR: 62–77) and a male predominance with a male to female ratio of approximately 4:1. Intravesical chemotherapy and BCG treatments before PD-L1 testing were administered to 56 (7%) and 106 (14%) patients, respectively. Of the patients, 266 (35%) presented with metastases to the lymph node only (17%) or to distant organs (18%) and 115 (15%) received systemic chemotherapy prior to PD-L1 testing. 500 (66%) and 261 (34%) patients showed organ confined (stage I/II) and non-organ confined (stage III/IV) disease, respectively. Most of the specimens for the PD-L1 testing were obtained from lower tract (83%) including the bladder and surgical procedures for UC of the bladder (82%; TUR, 55%; cystectomy, 27%). In the entire cohort, a total of 213 (28%) patients showed positive PD-L1 expression (Table 1). The positive PD-L1 expression group had a lower frequency of intravesical BCG before PD-L1 tesing, more specimens obtained from cystectomy, more non-organ confined disease, and higher tumor grade compared to the negative group (all *P*-values < 0.05, listed in Table 1).

**Table 1:** Baseline characteristics of the entire cohort (*n* = 761) and comparative analysis by PD-L1 expression status.

**Variables**	**Total (*n* = 761)**	**Negative PD-L1 (<5%) (*n* = 548, 72%)**	**Positive PD-L1 (≥5%) (*n* = 213, 28%)**	***P*-value**
**Clinical parameters**				
Age at surgery, years, median (IQR) <70 ≥70	70 (62–77) 369 (48%) 392 (52%)	258 (47%) 290 (52%)	111 (52%) 102 (48%)	0.23
Sex, *n* (%)				
Male Female	619 (81%) 142 (19%)	440 (80 %) 108 (20%)	179 (84%) 34 (16%)	0.25
Smoking status at diagnosis, *n* (%)				
No smoking history Smoking history (ex-or current) Missing	318 (42%) 323 (42%) 120 (16%)	246 (45%) 248 (45%) 53 (10%)	72 (34%) 74 (34%) 67 (32%)	<0.01
Intravesical chemotherapy prior to VENTANA, *n* (%)				
No Yes	705 (93%) 56 (7%)	507 (93%) 41 (7%)	198 (93.0%) 15 (7.0%)	0.88
Intravesical BCG prior to VENTANA, *n* (%)				
No Yes	655 (86%) 106 (14%)	463 (84%) 85 (16%)	192 (90%) 21 (10%)	0.04
Systemic chemotherapy prior to VENTANA, *n* (%)				
No Yes	646 (85%) 115 (15%)	471 (86%) 77 (14%)	175 (82%) 38 (18%)	0.21
Metastasis, *n* (%)				
No Lymph node only Distant (lung, liver, bone, colon, etc.)	495 (65%) 127 (17%) 139 (18%)	368 (67%) 81 (15%) 99 (18%)	127 (59%) 46 (22%) 40 (19%)	0.06
**VENTANA related parameters**				
Specimen type, *n* (%)				
TUR (pre-BCG) TUR (post-BCG) Cystectomy (radical or partial) Nephroureterectomy or ureterectomy Biopsy for primary lesion Biopsy for metastatic lesion	344 (45%) 72 (9%) 203 (27%) 100 (13%) 23 (3%) 19 (3%)	274 (50%) 64 (12%) 97 (18%) 74 (13%) 23 (4%) 16 (3%)	70 (33%) 8 (4%) 106 (50%) 26 (12%) 0 (0%) 3 (1%)	<0.01
Organ type, *n* (%)				
Lower tract (bladder or urethra) Upper tract (renal pelvis or ureter) Metastatic site	633 (83%) 109 (14%) 19 (3%)	449 (82%) 83 (15%) 16 (3%)	184 (86%) 26 (12%) 3 (2%)	0.27
Operation type, *n* (%)				
TUR Cystectomy (radical or partial) Nephroureterectomy or ureterectomy Biopsy	416 (55%) 203 (27%) 100 (13%) 42 (5%)	338 (62%) 97 (18%) 74 (13%) 39 (7%)	78 (37%) 106 (50%) 26 (12%) 3 (1%)	<0.01
Pathologic stage, *n* (%)				
Organ confined (stage I/II) Non-organ confined (stage III/IV)	500 (66%) 261 (34%)	384 (70%) 164 (30%)	116 (55%) 97 (45%)	<0.01
Pathologic grade, *n* (%)				
Low grade High grade	101 (13%) 660 (87%)	94 (17%) 454 (83%)	7 (3%) 206 (97%)	<0.01
Time from tissue acquisition to VENTANA, *n* (%)				
<1 month ≥1 month	501 (66%) 260 (34%)	364 (66%) 184 (34%)	137 (64%) 76 (36%)	0.61

*PD-L1, programmed cell death-ligand 1; BCG, bacillus Calmette–Guerin; TUR, transurethral resection; IC, immune cells*.

### Factors Related to Positive PD-L1 Expression in UC Patients

**Figure 1:**
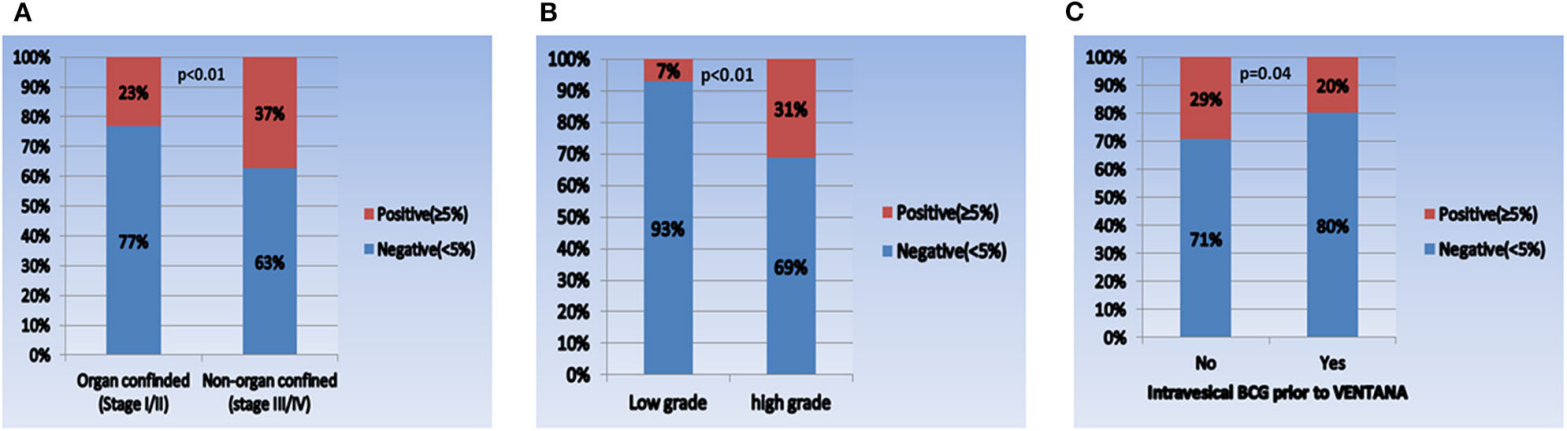
Comparison of PD-L1 expression in each subgroup. **(A)** Pathologic tumor stage (I/II vs. III/IV), **(B)** pathologic tumor grade (low vs. high), and **(C)** intravesical BCG prior to VENTANA test (no vs. yes).

**Figure 2:**
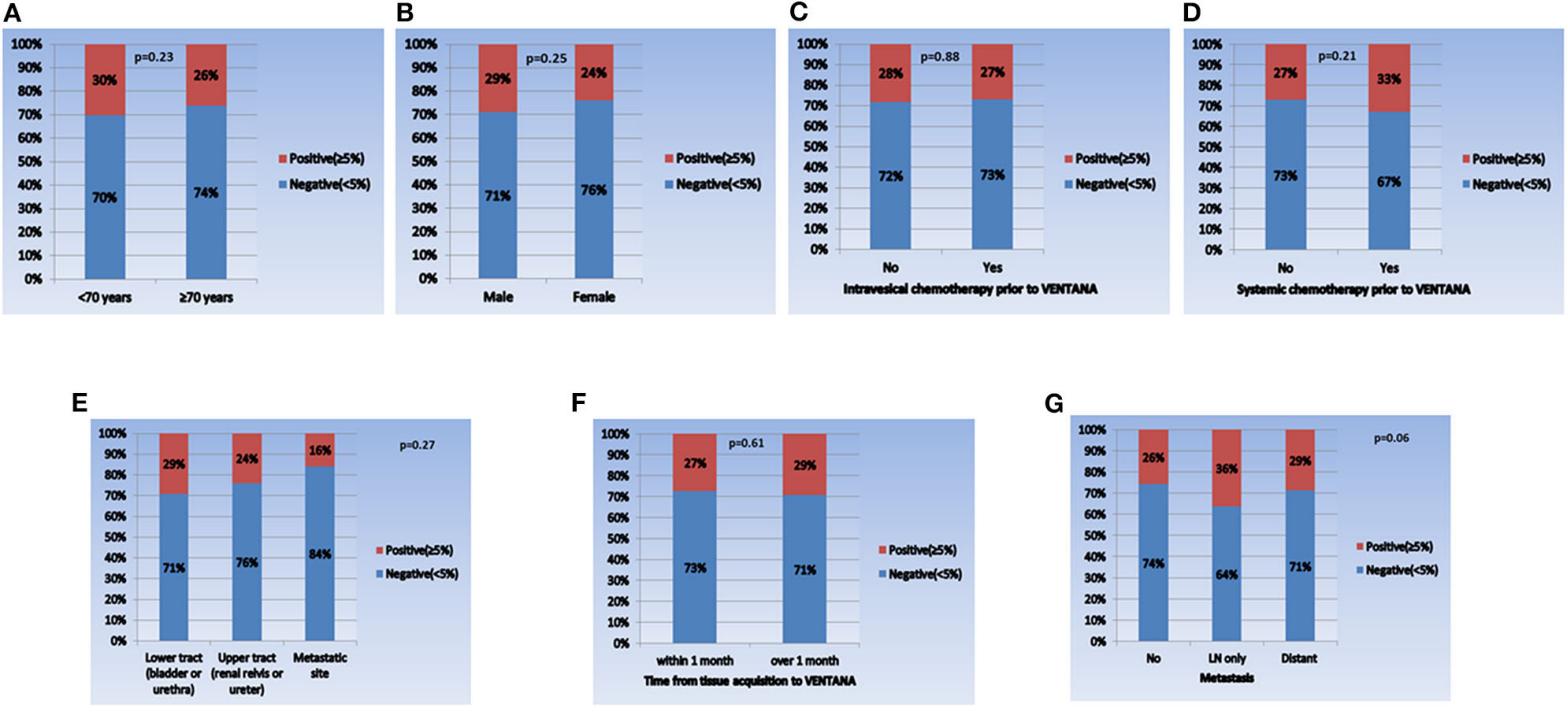
Comparison of PD-L1 expression in each subgroup. **(A)** Age (<70 years vs. **≥**70 years), **(B)** sex (male vs. female), **(C)** intravesical chemotherapy prior to VENTANA (no vs. yes), **(D)** systemic chemotherapy prior to VENTANA (no vs. yes), **(E)** specimen acquisition site (lower tract vs. upper tract vs. metastatic site), **(F)** time from tissue acquisition to VENTANA test (<1 month vs. ≥1 month), and **(G)** metastatic status (no vs. lymph node only vs. distant organ metastasis).

When comparing the PD-L1 expression level between subgroups, the rate of positive PD-L1 expression differed according to tumor stage, tumor grade, and intravesical BCG treatment prior to PD-L1 testing (Figure 1). Positive PD-L1 expression was frequently observed in non-organ confined disease (stage III/IV), high grade tumors, and in samples from patients without intravesical BCG treatment before PD-L1 testing. Whereas, there were no significant correlations between PD-L1 expression level and other parameters, including age (as a binary variable), sex, metastatic status, intravesical or systemic chemotherapy, organ type for PD-L1 testing, and time from tissue acquisition to PD-L1 testing (Figure 2).

To determine the related factors with positive PD-L1 expression, unadjusted and adjusted logistic regression analyses using two separate models (including age as a continuous or categorical variable) were performed (Table 2). The results revealed that increased age (odds ratio [OR] 0.98, 95% confidence interval [CI] 0.97–0.99), intravesical BCG prior to PD-L1 testing (OR 0.57, 95% CI 0.34–0.96), upper tract origin (OR 0.49, 95% CI 0.29–0.81), non-organ confined disease (OR 2.47, 95% CI 1.41–2.93), and high tumor grade (OR 5.31, 95% CI 2.38–11.83) were the independent factors of positive PD-L1 expression. In other words, several clinical factors, including younger age, no intravesical BCG treatment prior to PD-L1 testing, samples obtained from the lower urinary tract including the bladder, advanced stage and high grade tumors, were significantly associated with positive PD-L1 expression.

**Table 2:** Unadjusted and adjusted logistic regression analyses for the related factors with positive PD-L1 expression.

	**Unadjusted analysis**		**Adjusted analysis including age as continuous variable**		**Adjusted analysis including age as categorical variable**	
**Variables**	**Unadjusted OR (95% CI)**	***P*-value**	**Adjusted OR (95% CI)**	***P*-value**	**Adjusted OR (95% CI)**	***P*-value**
**Age (continuous)**	**0.98 (0.97–1.00)**	**0.05**	**0.98 (0.97–0.99)**	**0.04**		
Age (<70 vs. ***≥70***)	0.82 (0.60–1.12)	0.21			0.78 (0.56–1.09)	0.15
Sex (male vs. ***female***)	0.77 (0.51–1.18)	0.23	0.66 (0.42–1.02)	0.06	0.67 (0.43–1.05)	0.08
Intravesical chemotherapy (no vs. ***yes***)	0.94 (0.51–1.73)	0.83	1.61 (0.77–3.33)	0.20	1.59 (0.77–3.28)	0.21
**Intravesical BCG (no vs**. ***yes*****)**	**0.59 (0.36–0.99)**	**0.04**	**0.57 (0.34–0.96)**	**0.03**	**0.55 (0.33–0.94)**	**0.03**
Systemic chemotherapy (no vs. ***yes***)	1.33 (0.87–2.03)	0.19	1.14 (0.72–1.80)	0.57	1.14 (0.72–1.81)	0.56
Organ type (ref. lower tract)						
**Upper tract**	0.76 (0.48–1.23)	0.26	**0.49 (0.29–0.81)**	**0.006**	**0.48 (0.29–0.81)**	**0.006**
Metastatic site	0.46 (0.13–1.59)	0.22	0.34 (0.09–1.22)	0.10	0.33 (0.09–1.21)	0.10
**Stage (organ confined vs**. ***non-organ confined*****)**	**1.96 (1.41–2.71)**	**<0.01**	**2.04 (1.41–2.93)**	**<0.01**	**2.00 (1.39–2.88)**	**<0.01**
**Grade (low grade vs**. ***high grade*****)**	**6.09 (2.78–13.36)**	**<0.01**	**5.31 (2.38–11.83)**	**<0.01**	**5.30 (2.38–11.80)**	**<0.01**
Time from tissue acquisition to VENTANA	1.10 (0.79–1.53)	0.58	0.89 (0.62–1.28)	0.53	0.89 (0.62–1.28)	0.54
(month) (<1 vs. ***≥1***)						

*PD-L1, programmed cell death-ligand 1; IC, immune cells; BCG, bacillus Calmette–Guerin*.

## Discussion

PD-L1 expression has been extensively studied to assess their potential for predicting response to ICI therapy in metastatic UC. In the phase II IMvigor210 trial, cohort 2 on atezolizumab for second-line use, IC2/3 PD-L1 expression (defined as ≥5%) was associated with a higher overall response rate of 26% compared with 15% in all patients (4). This trend was also observed in several ICI related clinical trials in metastatic UC (2, 5, 7). However, results from other clinical trials showed no significant correlation between PD-L1 expression and response to ICI in patients with metastatic UC (3, 6, 8, 9). For example, data from phase III trials, such as KEYNOTE-045 and IMvigor 211, showed poor correlation of response to PD-1 and PD-L1 inhibitors with PD-L1 status (8, 9). In addition to PD-L1, several biomarkers such as tumor mutation burden, molecular subtyping, and immune gene expression profile, have been extensively studied as the factors that can help predict the response to ICI (11–14, 28). Besides, it was recently reported that several clinical factors, such as low neutrophil-to-lymphocyte ratio (<5) and lack of visceral metastasis, may have predictive utility for clinical benefit, defined as any objective reduction in tumor size, to ICI (29). These conflicting results describing the association between PD-L1 expression and patient response may be attributed to the heterogeneity of the four different IHC assays used for measuring PD-L1 expression. First, clinical trials with pembrolizumab and nivolumab use the Dako assay with the 22C3 and 28-8 detection antibodies, respectively (12, 24, 25). On the other hand, clinical trials with atezolizumab and durvalumab use the VENTANA assay with SP142 and SP263 antibodies, respectively (12, 24, 25). Second, the cell populations assayed for scoring PD-L1 expression differ for each ICI (12, 24, 25). In the IMvigor trials of atezolizumab, PD-L1 expression was scored using ICs (3, 4, 9), whereas the CheckMate trial for nivolumab used TCs to score PD-L1 expression (6). Pembrolizumab, durvalumab, and avelumab clinical trials scored PD-L1 expression using combined TCs + ICs, CPS (5, 7, 8). Third, different cutoff values are applied to define positive PD-L1 expression for each ICI. In the case of atezolizumab, PD-L1 expression ≥5% was defined as positive PD-L1 expression (3, 4, 9). The definition of positive PD-L1 expression was PD-L1 ≥1% for nivolumab, ≥10% for pembrolizumab, ≥25% for durvalumab, and ≥5% for avelumab (5–8). In the end, the lack of IHC assay standardization can cause variability in study results and hamper understanding of the role of PD-L1 as a biomarker.

In this study, in an attempt to minimize the influence of the heterogeneities between PD-L1 IHC assays, the VENTANA test was selected as the only IHC assay for measuring PD-L1 expression. Because the primary objective of this study was to identify PD-L1 expression status in UC regardless of atezolizumab use, UC specimens at all stages obtained through a variety of surgical procedures were included in the analysis. Generally, positive PD-L1 expression was observed in 28% (213/761) of all UC patients, which is similar to previous reports (15, 27). In particular, positive PD-L1 expression was more common in non-organ confined stage III/IV disease (37%) and high-grade tumors (31%) than in organ-confined stage I/II disease (23%) and low grade tumors (7%). These correlations between high PD-L1 expression and unfavorable pathological features (advanced stage and high grade tumor) were also identified in prior studies (15, 16). Meanwhile, positive PD-L1 expression was more common in patients that had received no intravesical BCG (29%) than in those that had (20%). This finding contradicts previous studies, which reported higher PD-L1 expression in BCG non-responders (including BCG relapsing tumors) than in BCG responders in non-muscle invasive bladder cancer (NMIBC) (30, 31). This discrepancy may be due to a variety of reasons. First, a history of BCG therapy before tissue acquisition for VENTANA does not necessarily indicate BCG unresponsiveness. Second, unlike the previous studies, which included only NMIBC patients, our study population consisted of UC patients at all disease stages. In the subgroup analysis of NMIBC patients only (*n* = 284), the positive PD-L1 expression rate was not significantly different according to BCG therapy (pre-BCG: 10%, post-BCG: 6%, *P* = 0.44, Supplementary Figure 1), which is consistent with the result from the previous study (31). As mentioned earlier, because PD-L1 as a tumor marker has dynamic properties that are influenced by various factors, we tried to evaluate the related factors for PD-L1 expression. We found that advanced tumor stage (stage III/IV), high tumor grade, younger age, no intravesical BCG, and lower tract specimen significantly correlated with positive PD-L1 expression. In summary, our study may provide meaningful information on the overall aspects of PD-L1 expression and the factors related to its expression in Korean UC, which has not been reported to date.

The current study has some limitations. First, there may be inherent biases resulting from its multi-institutional and retrospective features. For instance, there was no standardization of the surgical procedures, including TUR, cystectomy, nephroureterectomy and biopsy, which were performed to obtain the UC specimens for the PD-L1 assays in each institution. In addition, there were a different number of patients from each institution, ranging from a minimum of 8 to a maximum of 297 per institution. As a result, the data from the institution enrolling the largest number of patients may have had a stronger influence on the overall study results than that from other institutions. However, these factors can also be interpreted as strengths of this study because they reflect real-world clinical experiences and help to generalize the results. Finally, the present study considered PD-L1 expression in UC of all stages, irrespective of ICI use. Thus, PD-L1 has not been evaluated as a biomarker for predicting response to and prognosis following ICI treatment. However, the study showed that the rate of positive PD-L1 expression was significantly higher in advanced stage and high-grade tumors, indicating that PD-L1 expression may be also related to prognosis. To elucidate this point, further follow-up studies will be needed to assess the relationship between PD-L1 expression and response to treatment and clinical outcomes for ICI therapy in the metastatic UC setting.

## Conclusion

Positive PD-L1 expression, defined as IC coverage ≥5% as evaluated by the VENTANA test, was found in 28% of Korean UC patients, and multiple factors, including younger age, no intravesical BCG, lower tract specimen, advanced tumor stage, and high-grade tumor correlated with positive PD-L1 expression. To establish the utility of PD-L1 as a biomarker, further follow-up clinical trials will be needed and should consider adjustment for some factors, including intravesical BCG treatment, tumor stage, and grade in the analysis.

## Data Availability Statement

The raw data supporting the conclusions of this article will be made available by the authors, without undue reservation.

## Ethics Statement

The studies involving human participants were reviewed and approved by the Institutional Review Board of each participating institution, including Dongguk University Ilsan Medical Center. Written informed consent for participation was not required for this study in accordance with the national legislation and the institutional requirements.

## Author Contributions

HSK, WSH, WSJ, SIJ, DHL, WS, JHK, JYK, HKH, SYC, IHC, TC, BCJ, SHK, HKS, and SSJ: conception and design. HSK, WSH, WSJ, SIJ, DHL, WS, JHK, JYK, HKH, SYC, IHC, TC, SHK, and HKS: acquisition of data. HSK and HKS: analysis and interpretation of data. HSK, SHK, and HKS: writing or revision of the manuscript. HKS and SSJ: administrative, technical, or material supports, and supervision. All authors contributed to the article and approved the submitted version.

## Conflict of Interest

The authors declare that the research was conducted in the absence of any commercial or financial relationships that could be construed as a potential conflict of interest.

## Supplementary Material

Supplementary Figure 1 Comparison of PD-L1 expression according to BCG therapy in patients with non-muscle invasive bladder cancer (*n* = 284).
