# Supplementary figures and images for: Programmed Cell Death-Ligand 1 Expression Status in Urothelial Carcinoma According to Clinical and Pathological Factors: A Multi-Institutional Retrospective Study
Source: Front Oncol. 2020 Oct 2;10:568809. doi: 10.3389/fonc.2020.568809 (PMC7562813; doi:10.3389/fonc.2020.568809)

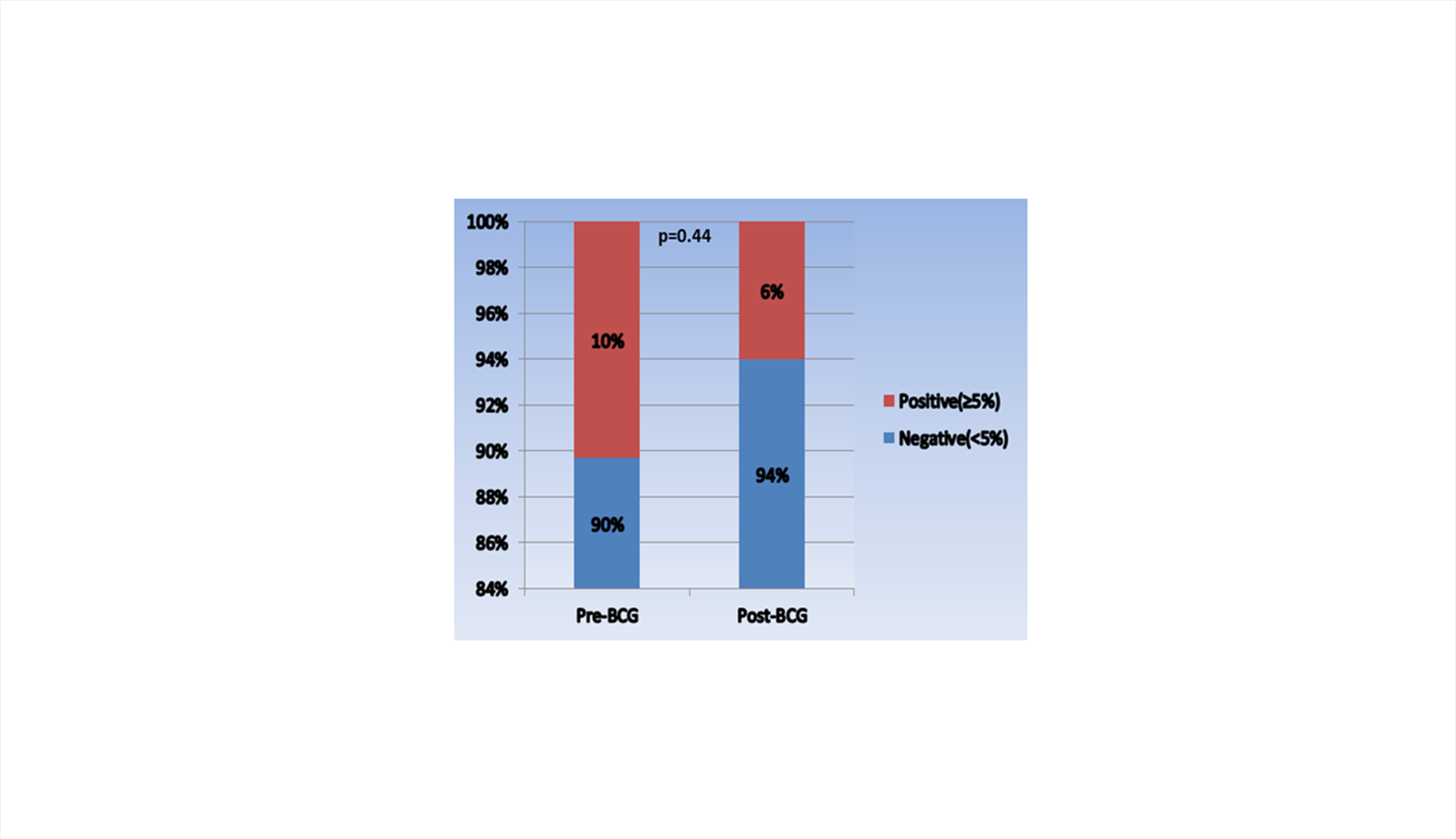

Supplement: Supplementary Figure 1 — Comparison of PD-L1 expression according to BCG therapy in patients with non-muscle invasive bladder cancer (n = 284). [file Image_1.TIF]
